# Estradiol Replacement Modulates Synaptic Transmission‐Related Proteins in the Hippocampus of Ovariectomized Rats: An Exploratory Proteomic Approach

**DOI:** 10.1111/jnc.70536

**Published:** 2026-08-14

**Authors:** Amanda Paula Pedroso, Adriana Pereira Souza, Guilherme Araújo Câmara, Meira Maria Forcellini Machado, Valter Tadeu Boldarine, Lila Missae Oyama, Mônica Marques Telles, Alexandre Keiji Tashima, Eliane Beraldi Ribeiro

**Affiliations:** ^1^ Department of Physiology Universidade Federal de São Paulo, Escola Paulista de Medicina São Paulo São Paulo Brazil; ^2^ Post‐Graduate Program in Chemical Biology, Institute of Environmental Sciences, Chemical and Pharmaceutical Universidade Federal de São Paulo Diadema São Paulo Brazil; ^3^ Department of Biochemistry Universidade Federal de São Paulo, Escola Paulista de Medicina São Paulo São Paulo Brazil

**Keywords:** estrogen, menopause, metabolism, proteome

## Abstract

The loss of ovarian hormones in postmenopause influences cognition, emotion and energy homeostasis, processes integrated by the hippocampus. The mechanisms by which estradiol influences this area have not been fully understood. The present study aimed at investigating the effects of estradiol on the rat hippocampus proteome of ovariectomy‐induced menopause either followed by estradiol replacement or not. Eighteen 3‐month‐old female Wistar rats were either ovariectomized or sham operated and fed with standard chow for 3 months. A subgroup of ovariectomized rats received estradiol replacement. The hippocampi were processed using data independent acquisition MS‐based proteomics and differentially expressed proteins were submitted to bioinformatics analysis for a pathway‐based functional understanding of the estradiol effects. Proteomic analysis revealed that 49 hippocampal proteins were modulated by ovariectomy and estradiol replacement. Functional analysis of the differentially expressed proteins revealed the enrichment of terms related to energy metabolism (comprising glycolysis/gluconeogenesis, pyruvate metabolism, oxidative phosphorylation, and response to oxidative stress) and neuron projection (comprising cytoskeleton organization, regulation of vesicle‐mediated transport, and modulation of chemical synaptic transmission). The present dataset indicates that, at the hippocampus, estradiol affects mitochondrial dynamics, lipid metabolism and intracellular trafficking machinery and influences dendritic and synaptic transmission and brain plasticity. Some alterations observed in proteins have not yet been described in the hippocampus in the context of menopause and estradiol replacement. These data provide new insights into the mechanisms involved with menopause effects and may help future studies related to drug development for the prevention and treatment of postmenopause associated symptoms.

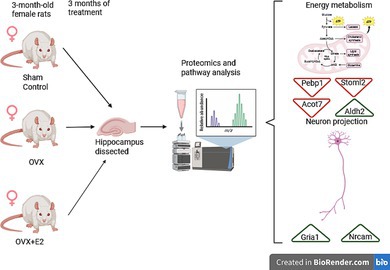

## Graphical abstract

The estrogen deficiency in the hippocampus influences cognitive functions and control of food intake. Hippocampi of female rats, either control or ovariectomized, with or without estrogen replacement, were submitted to LC–MS/MS and analysis of pathway‐based functional roles of proteins affected by estrogen deficiency and replacement. Results showed altered proteins in mitochondrial dynamics, lipid metabolism and intracellular trafficking machinery and influences dendritic and synaptic transmission and brain plasticity pathways, some of which has not yet been described in the hippocampus in the context of estrogen deficiency and replacement. These data provide new insights into the mechanisms involved with estrogen deficiency consequences.

**Abbreviations**
AGEs: advanced glycation end products
CNS: central nervous system
E2: estradiol
GO: gene ontology
GSTs: glutathione S‐transferases
HT: hormone therapy
KEGG: Kyoto Encyclopedia of Genes and Genomes
Ovx: ovariectomy
OXPHOS: oxidative phosphorylation
PDC: pyruvate dehydrogenase complex
Prdx: peroxiredoxins
T2D: type 2 diabetes
VMS: vasomotor symptoms

## 1 Introduction

The loss of ovarian‐derived hormones that characterizes postmenopause induces metabolic and phenotypical changes that may impact women's health and well‐being from midlife to old age. Abdominal fat accumulation, insulin resistance, type 2 diabetes (T2D), and cardiovascular diseases have been associated with postmenopause (Davis et al. 2015). Ovarian failure also induces symptoms related to central nervous system (CNS) alterations, including vasomotor symptoms (VMS), memory complaints, migraine, sleep disorders, depression, and anxiety (Weber et al. 2012; Monteleone et al. 2018; Naufel et al. 2018; Naufel et al. 2021). Although estrogen‐containing hormone therapy (HT) is the most effective treatment for menopausal VMS, clinical trials have evidenced diverse neurological effects, mainly those related to cognition, depending on menopause duration, time of treatment, HT regimens, and prior vulnerability (McCarrey and Resnick 2015; Brinton 2016; Thaung Zaw et al. 2018).

At the CNS, the hippocampus orchestrates essential processes such as learning and memory consolidation and takes part in the regulation of emotion, anxiety, stress, and the reward system. Clinical studies have reported an association of postmenopause symptoms and HT with altered hippocampal volumes, activation state, and bilateral connectivity (Ha et al. 2007; Berent‐Spillson et al. 2010; Goto et al. 2011; Jacobs et al. 2016; Maki et al. 2020). Bilateral ovariectomy in rats induced depression and memory impairments along with hippocampal volume reduction, neuronal loss, and increased autophagy (Su et al. 2012; Fang et al. 2018; Pandey et al. 2020).

It is worth pointing out that through the integration of emotion and cognition processes with body energy status signals, the hippocampus also participates in brain circuitries regulating appetite and feeding behavior (Kanoski and Grill 2017). In fact, the reciprocal influence between cognitive and emotional factors and eating behavior has already been emphasized (Spencer et al. 2017; AlAmmar et al. 2020). Not surprisingly, peripheral and brain‐derived chemical messengers showing a key role in energy homeostasis (such as insulin, leptin and serotonin) also modulate hippocampal function, and the crosstalk of these molecules with estrogen has been indicated (Amin et al. 2005; Clegg et al. 2006; Kanoski et al. 2011; Somogyi et al. 2011; Suarez et al. 2019). We have previously shown that, although estradiol (E2) replacement failed to normalize ovariectomy‐induced obesity, it did improve peripheral insulin sensitivity and decreased leptin levels (Boldarine et al. 2019, 2020, 2021), supporting results observed in postmenopausal women (Margolis et al. 2004). Similarly, E2 replacement has also been able to restore brain insulin sensitivity in ovariectomized rats, as long as these animals were not high‐fat diet‐fed (Pratchayasakul et al. 2014). A study from our group has previously demonstrated that hypoestrogenism affected the hippocampal serotonin receptor system (Machado et al. 2021).

Importantly, several pieces of evidence have indicated that E2 affects hippocampal synaptic plasticity (Spencer‐Segal et al. 2012; Bredemann and McMahon 2014; Hasegawa et al. 2015), which influences its function and may induce behavior changes. However, the cellular and molecular mechanisms by which E2 promotes these effects have not been fully understood. Considering the hippocampal function on the postmenopause‐associated symptoms and the influence of E2 at this brain region, the present study aimed at comprehensively investigating the effects of E2 on the rat hippocampus proteome using an animal model of ovariectomy‐induced menopause either followed by estradiol replacement or not.

## 2 Material and Methods

### 2.1 Study Design

Animal protocols were approved by the Research Ethics Committee of the Universidade Federal de São Paulo—UNIFESP (#2172030315), according to the ethics guidelines settled by the Brazilian Conselho Nacional de Controle de Experimentação Animal (CONCEA). The present dataset is part of our previously published investigation in which we analyzed the effects of ovariectomy, allied or not to the intake of high‐fat diet, on metabolic and behavioral parameters of rats (Boldarine et al. 2019). Animals provided by Cemib (Unicamp, Campinas, SP, Brazil) were kept 2 or 3 rats per cage, under controlled lighting (12 h light:12 h dark cycle, lights on at 6 am) and temperature (23°C ± 1°C) conditions with free access to water and balanced chow (Nuvilab CR‐1, Nuvital Nutrientes SA, Colombo, PR, Brazil) throughout the experimental period. Twelve‐week‐old female Wistar rats (RRID, Research Resource Identifier) were submitted to either sham operation (Sham, *n* = 6) or bilateral ovariectomy (Ovx, *n* = 12) under ketamine/xylazine anesthesia (66/33 mg/kg, ip). A subgroup of Ovx animals received estrogen replacement (Ovx + E2, *n* = 6) through a subcutaneous implantation of a tablet of 17β‐estradiol (0.25 mg/pellet, 90‐day release; Innovative Research of America, Sarasota, FL, USA). After the surgery, all animals received penicillin (60.000 U, i.m.) and ibuprofen (1 mg/kg body weight, v.o.), and additional daily ibuprofen doses were administered for 2 days. The daily release of estrogen (2.8 μg/day/90 days) yielded a daily dose range of 0.010–0.008 mg/Kg/day, considering the mean initial and final body weights of the rats during the 12 weeks of treatment (Boldarine et al. 2019, 2020).

Body weight and food intake were measured weekly throughout the experimental period. At the end of 12 weeks of treatment rats were anesthetized with thiopental (80 mg/kg, ip) and euthanized by decapitation after 24 h fasting, in order to have all animals in the same metabolic condition. Ovaries removal was confirmed by the uteri weight. The hippocampus was rapidly dissected, flash‐frozen in liquid nitrogen and stored at −80°C until further analysis.

In the same animal cohort used in the present study, we have previously investigated the alterations induced by ovariectomy and estradiol replacement in the retroperitoneal adipose tissue proteome, and details on body and serum parameters have already been published (Boldarine et al. 2020). Briefly, we have shown that ovariectomy decreased uterine weights and induced obesity, hyperleptinemia, and insulin resistance. Although estradiol replacement failed to normalize body weight and adiposity, it improved insulin sensitivity and decreased leptin levels (Boldarine et al. 2020).

### 2.2 Sample Preparation

Each hippocampus was homogenized in 250 μL of lysis buffer (8 M urea, 2 M thiourea, 70 mM dithiothreitol) and protease/phosphatase inhibitor cocktail (Thermo Scientific, Rockford, IL, USA) and then, samples were centrifuged (19 000 × g, for 30 min, at 4°C). Protein concentration in the supernatants was determined using a Bradford assay (BioAgency, Sao Paulo, SP, Brazil). Aliquots of 200 μg were diluted in 50 mM ammonium bicarbonate to a final volume of 60 μL, followed by incubation with 25 μL of 0.2% solution of RapiGest SF (Waters, Milford, MA, USA) (for 15 min, at 80°C), reduction with 100 mM dithiothreitol (for 30 min, at 60°C), and alkylation with 300 mM iodoacetamide (for 30 min, at room temperature). Protein digestion was performed using trypsin (Promega, Fitchburg, WI, USA) at a 1:100 (wt:wt) enzyme: protein ratio (overnight, at 37°C), followed by incubation with 10 μL of 5% trifluoroacetic acid (for 90 min, at 37°C) and centrifugation (19 000 × g, for 10 min, at 4°C). Supernatants were recovered, dried in a vacuum concentrator centrifuge (Eppendorf, Hamburg, HH, Germany), and stored at −80°C until mass spectrometry analysis.

### 2.3 LC–MS/MS and Database Search

Mass spectrometry analysis was performed as previously described, with minor modifications (Pedroso et al. 2017). Six independent hippocampal samples of each group were analyzed in triplicates (totaling 18 runs per group) on a nanoAcquity UPLC system coupled to a Synapt G2 HDMS Q‐TOF mass spectrometer (Waters). Samples (5 μL) were injected onto a trap column (nanoAquity C18 trap column Symmetry 180 μm × 20 mm, Waters) and then transferred by an elution gradient to an analytical column (nanoAcquity C18 BEH 75 μm × 250 mm, 1.7 mm, Waters). Buffer A (0.1% formic acid in water) and B (0.1% formic acid in acetonitrile) were used to generate a 7%–35% B elution gradient run over 115 min at a flow rate of 275 nL/min. Data were acquired in data‐independent mode (HDMSE), with ion mobility separation, in the m/z range of 50–2000 and in resolution mode. Collision energies were switched from low energy (4 eV) to acquire data from precursor ions to high energy (ramped from 19 to 45 eV) to acquire data from MS/MS fragments. The ESI source was operated in positive mode (capillary voltage of 3.0 kV, block temperature of 100°C, and cone voltage of 40 V). Glu‐fibrinopeptide B (500 fmol/mL in 50% acetonitrile, 0.1 formic acid; Waters) was infused at 500 nL/min every 30 s using a nanoLockSpray apparatus for external calibration.

Raw data were processed by ProteinLynx Global Server software version 3.0.1 (Waters) with database search against *Rattus norvegicus* sequences in the UniProtKB/Swiss‐Prot manually annotated database (www.uniprot.org, including 7996 entries, downloaded 06‐27‐2017). The following search parameters were used for protein identification: automatic precursor and fragment mass tolerance, 2 missed cleavage sites allowed for trypsin digestion, cysteine carbamidomethylation as fixed modification and methionine oxidation as variable modification. The protein identification criteria also included a minimum of 1 fragment ion per peptide, 5 fragment ions per protein and 2 peptides per protein. The false discovery rate was set at 4%. Label‐free quantitative assessments based on peptide intensities were performed by integrating the intensities of the three most intense peptides of each identified protein (Silva et al. 2005). The mass spectrometry proteomics data have been deposited to the ProteomeXchange Consortium (Deutsch et al. 2023) via the PRIDE partner repository (Perez‐Riverol et al. 2022) with the dataset identifier PXD040816. Results were exported into Excel files.

### 2.4 Proteomic Data Analysis

Data normalization was performed using the sum of all protein intensities. The average of technical replicates was used to calculate the protein intensity of each biological sample. To assess biological variation, only proteins identified in at least four out of six biological replicates per group were considered. The biological variation between groups is presented here as fold change, calculated as the ratio from the mean raw intensity values of every protein of each group.

Proteome data were log transformed and analyzed by one‐way ANOVA followed by Tukey HSD post hoc test. Statistical significance was set at *p* < 0.05. Multiple testing corrections were not performed to minimize false negative results (Pascovici et al. 2016). Statistical analysis was performed in IBM SPSS Statistics 21 Software (IBM, Armonl, NY, USA). The effect size of each variable comparison was calculated as [*ƞ* ^2^ = variance between groups/total variance] and the magnitude of the effect was categorized as follows: *ƞ* ^2^ = 0.02 indicates a small effect; *ƞ* ^2^ = 0.13 indicates a medium effect; *ƞ* ^2^ = 0.26 indicates a large effect (Fritz et al. 2012; Ialongo 2016). An additional exploratory false discovery rate (FDR) analysis was performed using the free online platform MetaboAnalyst (http://www.metaboanalyst.ca), following the same criteria adopted for the ANOVA followed by Tukey's HSD post hoc test. After FDR correction, only two proteins remained significantly altered (FDR < 0.05): Phosphatidylethanolamine‐binding protein 1 and Cytosolic acyl‐coenzyme A thioester hydrolase (Table S1). Both proteins had also been identified in the analysis described above.

The proteins differentially expressed among the groups were analyzed on the STRING online database (http://string‐db.org/), a bioinformatics tool which predicts interactions derived from several sources (coexpression analysis, evolutionary signals across genomes, literature text‐mining, and orthology‐based transfer of evidence across organisms) (Doncheva et al. 2019), and then imported into Cytoscape, an open source platform (https://cytoscape.org/) used to visualize functional protein‐association networks (Shannon et al. 2003). Interactions were considered using STRING software, using medium stringency (confidence score = 0.4). To gain insights into the biological properties related to the altered proteins, enrichment tests based on Gene Ontology (GO biological processes, GO cellular component, and GO molecular function), Kyoto Encyclopedia of Genes and Genomes (KEGG), and Reactome Pathways were conducted. Clustering was performed according to Community Cluster (GLay) Annotation Set using AutoAnnotate plugin (Kucera et al. 2016). GO terms were also examined using the online tool QuickGO (https://www.ebi.ac.uk/QuickGO/) (Huntley et al. 2015). Venn diagram and boxplots were carried out using BioVenn (https://www.biovenn.nl/) (Hulsen et al. 2008) and R version 4.0.3 (R Core Team 2024), respectively.

## 3 Results

### 3.1 Effects of Ovariectomy and Estradiol Replacement on Hippocampus Proteome

A total of 1554 proteins, each one identified by at least 2 peptides, comprised the female rat hippocampus data set. After application of an inclusion criterion for quantification (presence in at least 4 biological replicates per group), 345 hippocampal proteins were compared among the Sham, Ovx, and Ovx + E2 groups (Table S2). Modulation of 49 hippocampal proteins was detected among the groups.

**FIGURE 1:**
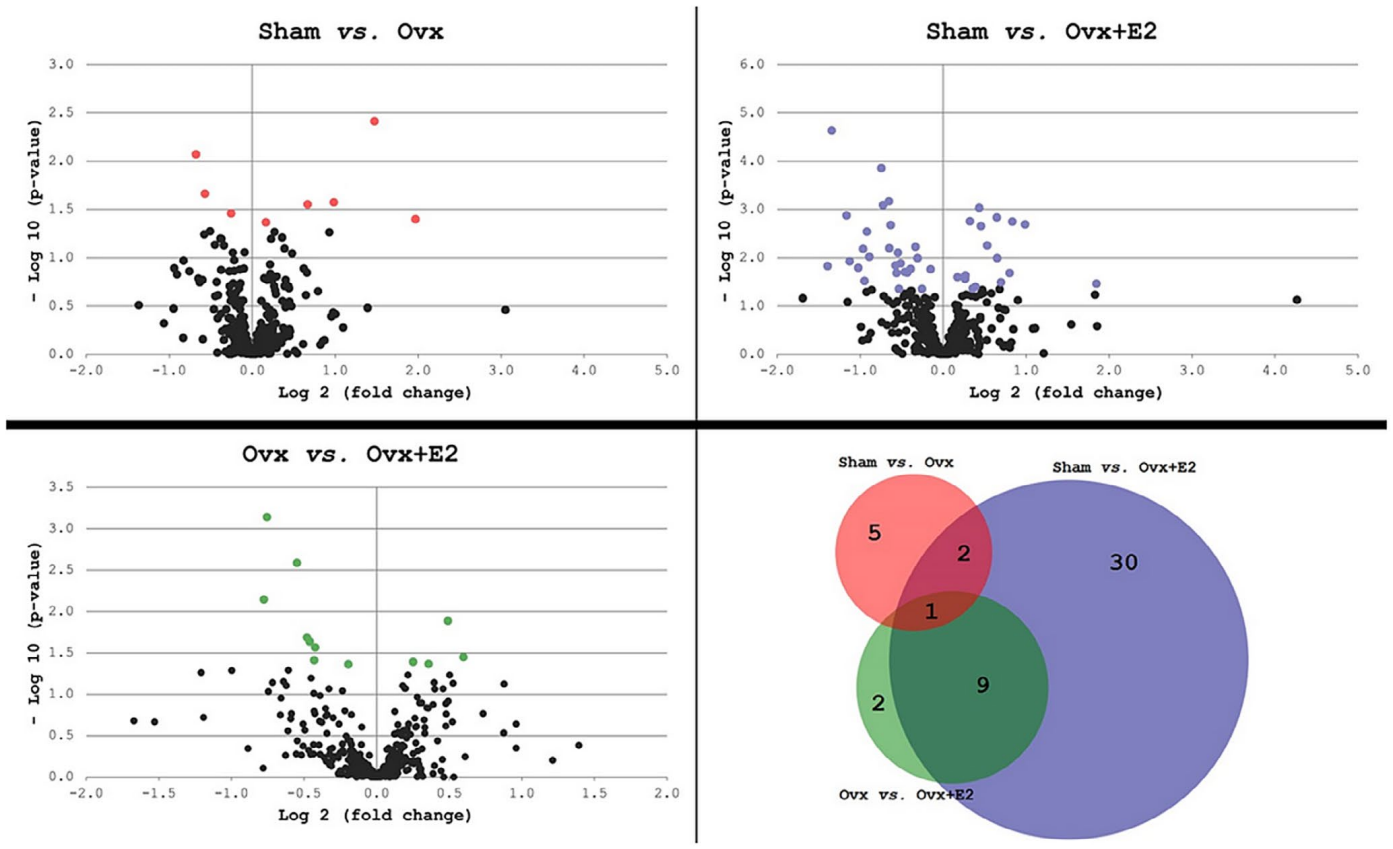
Volcano plots and Venn diagram representing proteins significantly altered in hippocampus dataset regarding the comparisons between Sham vs. Ovx (ovariectomized) groups (pink), Sham vs. Ovx + E2 groups (blue) and Ovx vs. Ovx + E2 groups (green). Fold change was calculated as the ratio from the mean raw values of protein intensity of each group; *p*‐value was based on Tukey HSD *post hoc* following ANOVA test.

The Ovx group showed decreased expression of 3 proteins and increased expression of 5 proteins in comparison to the Sham group. The Ovx + E2 group showed down‐regulation of 27 proteins and up‐regulation of 15 proteins in relation to the Sham group. Additionally, reduced expression of 8 proteins and raised expression of 4 proteins were observed in the Ovx + E2 group when compared to the Ovx group (Figure 1).

All the altered proteins, their gene symbols, as well as the fold change between groups are shown in Table 1. Additionally, database identifiers of the modulated proteins and a summary of their associated cellular location and biological process are shown in Table S3.

**TABLE 1:** Hippocampal proteins modulated by ovariectomy and estradiol replacement.

Protein | gene symbol	Fold change		
	Ovx/Sham	Ovx + E2/Sham	Ovx + E2/Ovx
Triosephosphate isomerase | Tpi1	0.93	0.90*	0.97
Phosphoglycerate mutase 1 | Pgam1	1.29	1.45**	1.12
Phosphoglucomutase‐1 | Pgm1	0.71	0.64**	0.91
L‐lactate dehydrogenase A chain | Ldha	0.78	0.65**	0.83
L‐lactate dehydrogenase B chain | Ldhb	0.74	0.69*	0.94
Pyruvate dehydrogenase E1 component subunit alpha | Pdha1	0.74	0.52*	0.70
Dihydrolipoyllysine‐residue acetyltransferase | Dlat	0.88	0.76*	0.87
Cytochrome c oxidase subunit 5A | Cox5a	1.14	1.31*	1.16
Stomatin‐like protein 2 | Stoml2	0.52	0.38*	0.73
Sideroflexin‐3 | Sfxn3	0.78	0.49*	0.63
D‐dopachrome decarboxylase | Ddt	1.12*	1.13*	1.01
Cytosolic acyl coenzyme A thioester hydrolase | Acot7	0.87	0.60***	0.68**
Aldehyde dehydrogenase | Aldh2	1.17	1.63*	1.39
Glutathione S‐transferase P | Gstp1	1.14	1.36***	1.19*
Peroxiredoxin‐2 | Prdx2	0.79	1.11	1.41*
Peroxiredoxin‐5 | Prdx5	0.90	0.80**	0.89
Phosphatidylethanolamine‐binding protein 1 | Pebp1	0.68*	0.39***	0.58**
Ubiquitin‐60S ribosomal protein L40 | Uba52	0.67	0.45**	0.67
Ubiquitin‐conjugating enzyme E2 N | Ube2n	0.92	0.73*	0.79
Sodium/potassium‐transporting ATPase subunit alpha‐3 | Atp1a3	1.17	1.20*	1.03
Sodium/potassium‐transporting ATPase subunit alpha‐4 | Atp1a4	3.92*	1.36	0.35
Plasma membrane calcium‐transporting ATPase 3 | Atp2b3	0.95	0.70*	0.74*
Plasma membrane calcium‐transporting ATPase 4 | Atp2b4	0.73	0.46*	0.63
ATP synthase subunit gamma | Atp5f1c	0.92	0.67*	0.73
V‐type proton ATPase 116 kDa subunit a isoform 1 | Atp6v0a1	0.81	0.53**	0.66
Alpha‐actinin‐1 | Actn1	1.97	3.61*	1.83
Spectrin alpha chain, non‐erythrocytic 1 | Sptan1	1.04	0.91	0.87*
Microtubule‐associated protein 6 | Map6	1.40	1.79**	1.28
Adenylyl cyclase‐associated protein 1 | Cap1	0.84*	0.81*	0.96
Tropomodulin‐2 | Tmod	0.64	0.55*	0.86
Profilin‐1 | Pfn1	1.39	1.75*	1.26
AP‐2 complex subunit beta | Ap2b1	0.86	0.84*	0.97
Endophilin‐A1 | Sh3gl2	1.02	0.61***	0.59***
Amphiphysin | Amph	0.80	0.51**	0.64
Synaptojanin‐1 | Synj1	1.59*	1.40	0.88
Vesicular glutamate transporter 1 | Scl17a7	1.31	1.99**	1.52*
Guanine nucleotide‐binding protein G (i) subunit alpha‐2 | Gnai2	2.78**	1.20	0.43
Calcium‐dependent secretion activator 1 | Cadps	0.92	0.68*	0.74
Calcium/calmodulin‐dependent protein kinase type II subunit gamma | Camk2g	0.63**	0.89	1.42
Calmodulin‐1 | Calm1	1.22	1.57**	1.28*
Glutamate receptor 1 | Gria1	1.98*	1.18	0.60
Leucine‐rich glioma‐inactivated protein 1 | Lgi1	1.02	0.74*	0.73*
Synaptophysin | Syp	0.83	0.54**	0.65
Carbonic anhydrase 2 | Car2	0.96	0.69**	0.72*
Neurochondrin | Ncdn	0.85	0.64***	0.75*
Neuronal cell adhesion molecule | Nrcam	1.20	1.57*	1.31
Neurofascin | Nfasc	1.04	1.21*	1.16
Alpha‐internexin | Ina	1.21	1.37*	1.14
Rab GDP dissociation inhibitor alpha | Gdi1	1.11	1.26**	1.13

*Note:* Fold change was calculated as the ratio from the mean raw values of protein intensity of each group. *p*‐value based on Tukey HSD *post hoc* following ANOVA test.

* *p* < 0.05.

** *p* < 0.01.

*** *p* < 0.001.

### 3.2 Protein–Protein Interaction Network and Enrichment Analysis

**FIGURE 2:**
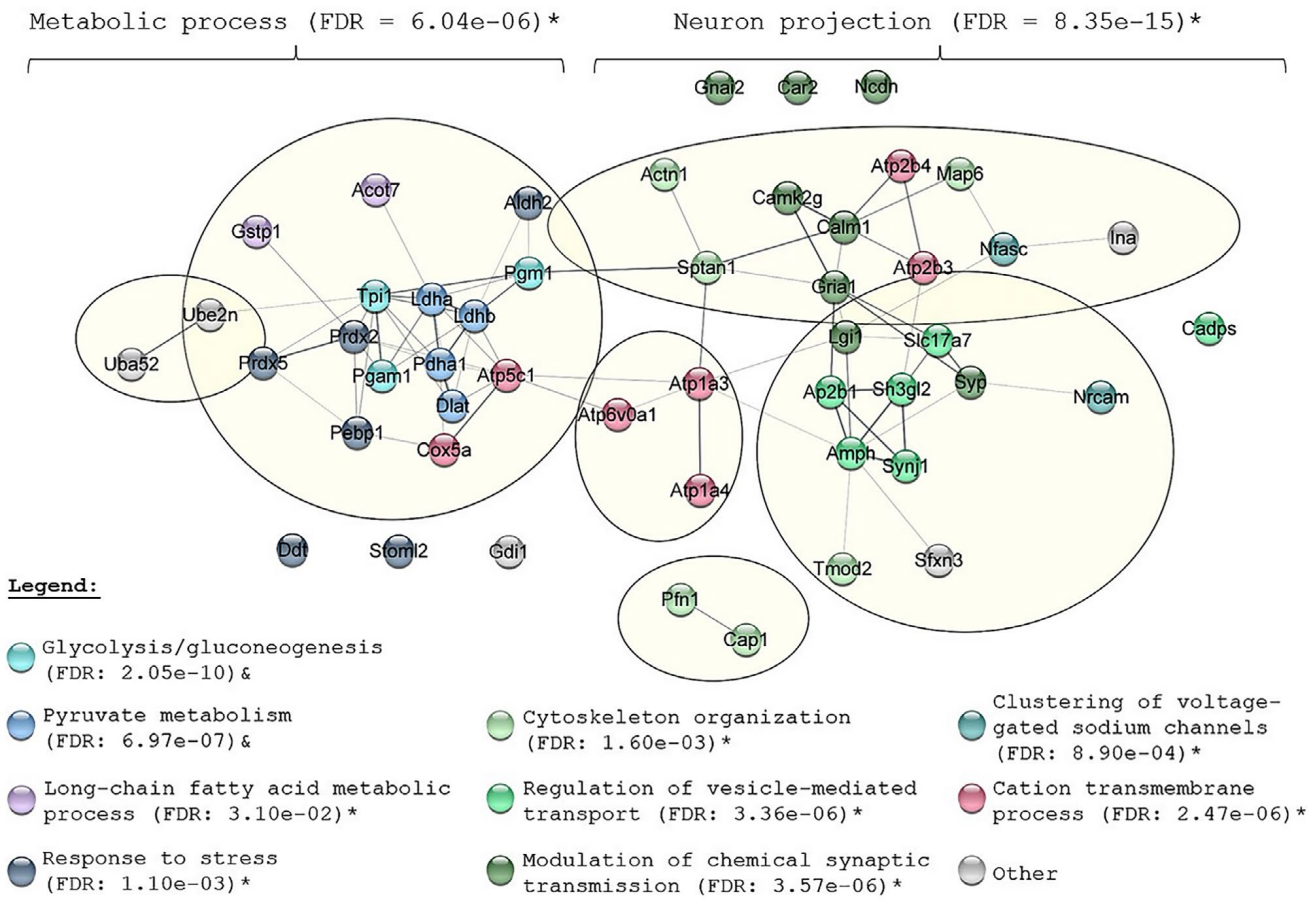
Interaction network of differentially expressed hippocampal proteins among the groups Sham, Ovx (ovariectomized), and Ovx + E2 (49 nodes; 79 edges; enrichment *p*‐value < 1.0e‐16). Clustering using community cluster algorithm grouped altered proteins in six main clusters, according to their function and connectivity. FDR (false rate discovery) values represent enrichment results based on the following annotation categories: *GO_Process; ^&^KEGG_Pathways. GO, Gene Ontology; KEGG, Kyoto Encyclopedia of Genes and Genomes.

**FIGURE 3:**
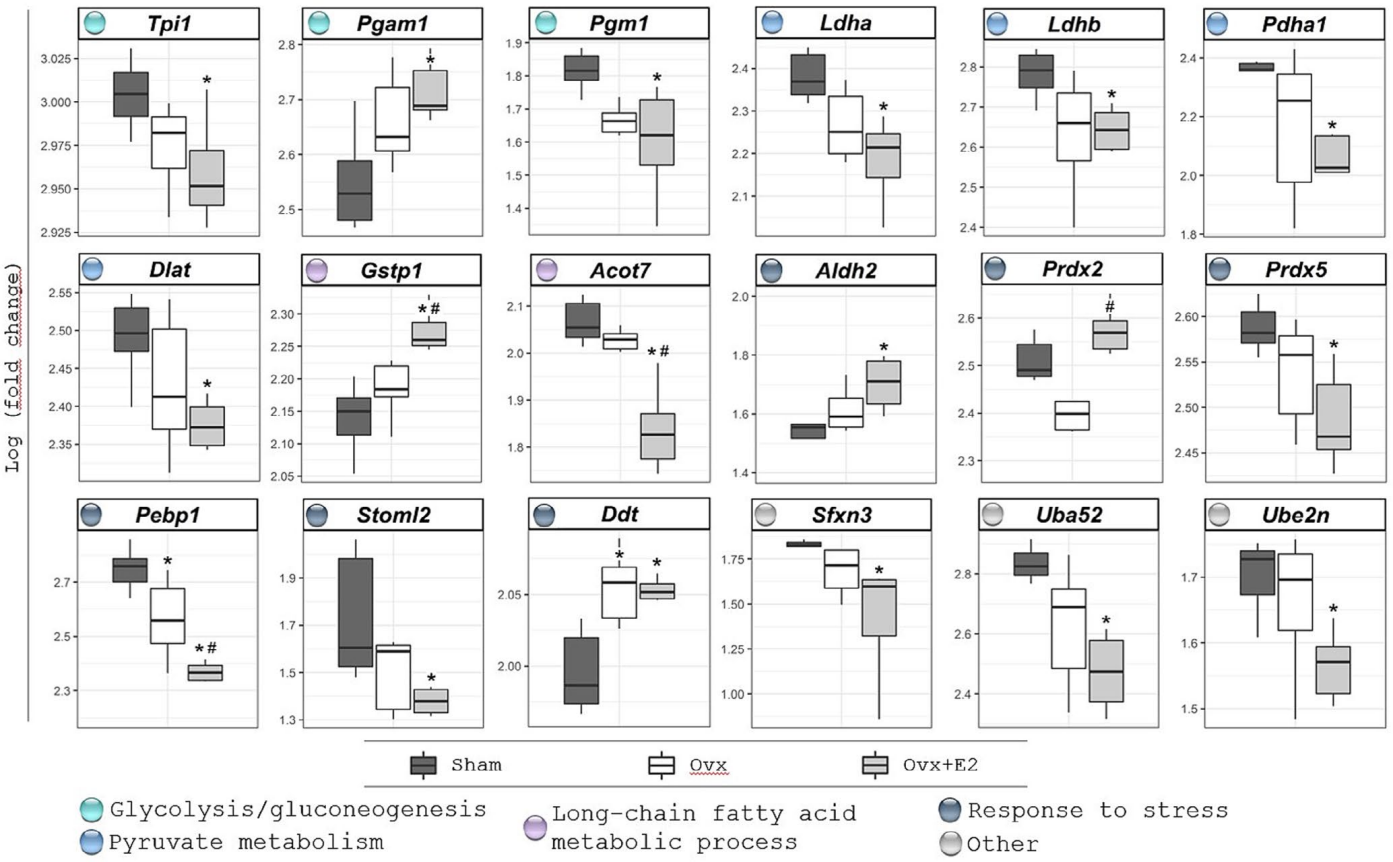
Boxplots of metabolic processes‐related proteins modulated by ovariectomy and estradiol replacement on the rat hippocampus. * vs. Sham; # vs. Ovx (ovariectomized).

**FIGURE 4:**
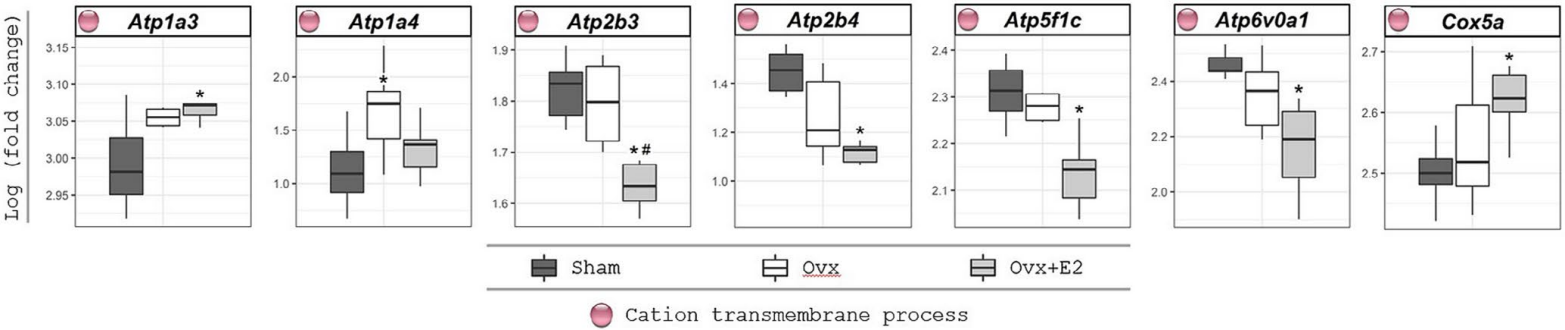
Boxplots of proteins showing ATPase activity, coupled to movement of substances, that were modulated by ovariectomy and estradiol replacement on the rat hippocampus. * vs. Sham; # vs. Ovx (ovariectomized).

**FIGURE 5:**
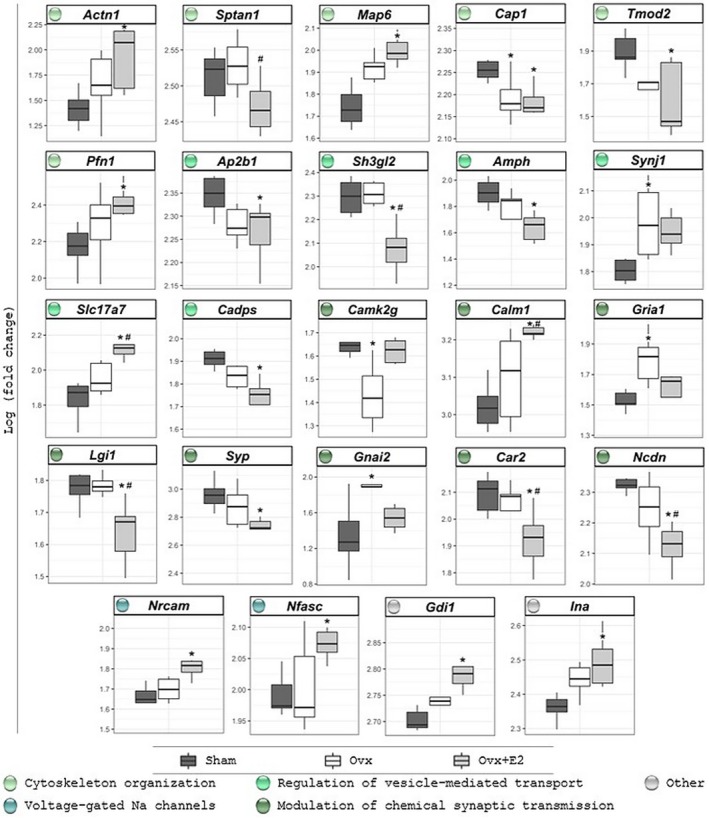
Boxplots of neuron projection‐related proteins modulated by ovariectomy and estradiol replacement on the rat hippocampus. * vs. Sham; # vs. Ovx (ovariectomized).

Modulated proteins were clustered based on direct (physical) and indirect (functional) interaction data available through the STRING database. This revealed that hippocampal proteins associated with ovariectomy and estradiol replacement showed more interactions with each other (49 nodes; 79 edges; enrichment *p*‐value < 1.0e‐16) than would be expected at random, indicating that these proteins are directly or indirectly connected. Clustering algorithm grouped proteins in six different clusters, according to their function and connectivity. Functional annotation analysis pointed out two main distinct subgroups among these clusters: metabolic process and neural projection (Figure 2). Table S3 shows detailed enrichment analysis of Gene Ontology (GO) terms, KEGG and Reactome pathways. Figures 3, 4, 5 show the boxplots indicating ms‐derived‐intensity variation of proteins modulated by ovariectomy and estradiol replacement on the rat hippocampus.

The subgroup metabolic process comprised 4 pathways/biological processes: glycolysis/gluconeogenesis (Tpi1, Pgam1 and Pgm1), pyruvate metabolism (Ldha, Ldhb, Pdha1 and Dlat), long‐chain fatty acid metabolic process (Gstp1 and Acot7), and response to stress (Aldh2, Prdx2, Prdx5, Pebp1, Stoml2 and Ddt). Ubiquitin cluster (Uba52 and Ube2n) was adjacent to this group (Figures 2 and 3). Although Sfxn3 did not show a direct interaction with proteins clustered in the metabolic process subgroup, it also showed significant differences among the experimental groups (Figure 3), and it is a mitochondrial component closely related to these pathways/processes.

Some altered proteins related to cation transmembrane process (Cox5a, Atp1a3, Atp1a4, Atp2b3, Atp2b4, Atp5c1, and Atp6v0a1) showed interactions with proteins belonging to both metabolic process subgroup and neuron projection subgroup, indicating a connection between them (Figures 2 and 4).

The neuron projection subgroup comprised 5 biological processes/cellular components: cytoskeleton organization (Actn1, Sptan1, Map6, Tmod2, Pfn1, and Cap1), regulation of vesicle‐mediated transport (Sh3gl2, Amph, Synj1, Slc17a7, Ap2b1, and Cadps), modulation of chemical synaptic transmission (Camk2g, Calm1, Gria1, Lgi1, Syp, Car2, Ncdn, and Gnai2), and axon initial segment (Nrcam and Nfasc) (Figures 2 and 5). Other proteins significantly modulated were Gdi1 (involved in vesicle‐mediated transport) and Ina (plays a role in neuronal morphogenesis) (Figures 3 and 5, respectively).

## 4 Discussion

Aiming at exploring the E2 function on the complex network influenced by the hippocampus, in the present study we used a label‐free proteomic approach to broadly analyze hippocampal proteins modulated as a result of ovariectomy alone or followed by E2 replacement. It is important to point out that the E2 effects in the CNS rely upon the administered E2 regimen. The E2 replacement dose used in the present study is compatible with the human dosage of the average transdermal replacement therapy for postmenopausal women (Hill et al. 2016; Nair and Jacob 2016). We have previously shown, in the same animal cohort studied in the present investigation, that ovariectomy induced obesity, insulin resistance, and hyperleptinemia, and that E2 replacement was able to improve peripheral insulin sensitivity and decrease leptin levels, although it failed to reverse the body weight gain (Boldarine et al. 2019). Although it has been previously indicated that E2 restored brain insulin sensitivity only in ovariectomized non‐obese rats while it had no effect in ovariectomized obese rats (Pratchayasakul et al. 2014), the present results evidence that a physiological dosage of E2 replacement does modulate hippocampal proteome in ovariectomy‐induced obese rats. The dataset evaluation indicated that the altered proteins are mainly related to energy metabolism and neuron projection, shedding light to the comprehension of the E2 impact on the postmenopause‐associated effects. It is important to point out that the results of the present study were obtained in fasted animals and that they may differ from the effects in an ad libitum condition.

### 4.1 Significant Enrichment of Terms Related to Metabolic Processes

The role of estrogen in the regulation of the brain bioenergetics system has been previously indicated (Nilsen et al. 2007). Accordingly, we observed that ovariectomy followed by E2 replacement induced changes in the expression of several proteins involved in energy metabolism and mitochondrial function. The glycolytic enzyme triosephosphate isomerase (Tpi1) is responsible for the interconversion between dihydroxyacetone phosphate (DHAP) and D‐glyceraldehyde‐3‐phosphate (GA3P), and the reduction of its activity has been associated with altered glucose metabolism and pyruvate production, cognitive dysfunction and aging‐induced neurodegeneration (Tajes et al. 2013; Zhao et al. 2013). Additionally, it has been indicated that Tpi1 deficiency induces the accumulation of DHAP, a precursor of both glycerolipids and glycerol‐ether lipids, which may favor derangements of lipid metabolism and impairment of the cellular redox homeostasis (Tajes et al. 2013). DHAP can also be converted into methylglyoxal, a toxic compound leading to the formation of advanced glycation end products (AGEs) (Tajes et al. 2013; Orosz et al. 2006). Intracellular production of AGEs has been shown to induce cell damage (Brownlee 2005), mitochondrial dysfunction (Akhter et al. 2021) and impairment of learning and memory processes (Lissner et al. 2021). In the present study, a significant decrease of Tpi1 expression in Ovx + E2 group in comparison to the Sham group was observed, suggesting disturbances of lipid metabolism and mitochondrial dysfunction induced by E2 replacement after ovariectomy. We also observed increased expression of phosphoglycerate mutase 1 (Pgam1), the glycolytic enzyme that catalyzes the conversion of 3‐phosphoglycerate to 2‐phosphoglycerate, in the Ovx + E2 group in relation to the Sham animals. This result may indicate an antioxidant response since reduction of neuronal damage induced by oxidative stress has been observed in hippocampal cell lines treated with Pgam1 (Kim et al. 2020).

It is important to point out that Tpi1 deficiency may also suggest altered pyruvate availability (Tajes et al. 2013). Pyruvate is the end product of glycolysis in the cytosol, and its transport to the mitochondrial matrix influences carbohydrate, amino acid, and fatty acid metabolism. Part of the glycolysis‐derived pyruvate is converted to lactate by lactate dehydrogenase (Ldh). According to the astrocyte‐neuron lactate shuttle hypothesis, under conditions of energy demand, glucose is metabolized in astrocytes and the astrocyte‐derived lactate is then metabolized in neurons through the TCA cycle (Pellerin and Magistretti 2004). Astrocytes express mainly the Ldha isoform, which preferably converts pyruvate into lactate, whereas the Ldhb isoform is expressed mainly in neurons and favors the conversion of lactate to pyruvate (Lam 2007). In the present study, decreased levels of both Ldha and Ldhb were observed in the Ovx + E2 group in comparison to the Sham group, indicating that E2 replacement after ovariectomy induced adjustments in pyruvate‐lactate metabolism and in the astrocyte‐neuron lactate transport, a mechanism necessary for long‐term memory formation (Suzuki et al. 2011). Interestingly, it has been shown that glycolysis blockage induced by an Ldha inhibitor prevented memory impairments in rats with streptozocin‐induced diabetes (Zhao et al. 2018).

The pyruvate dehydrogenase complex (PDC) has a key role in energy metabolism linking glycolysis to the TCA cycle through the oxidative decarboxylation of pyruvate to acetyl‐CoA. The PDC is composed of three enzymes: pyruvate dehydrogenase (component 1, Pdha1), dihydrolipoyllysine‐residue acetyltransferase (component 2, Dlat), and dihydrolipoyl dehydrogenase (component 3, Dld). Conditional Pdha1 knockout in the hippocampus has led to increased lactate levels, abnormal lactate transport, altered ultrastructure of mitochondria, and rough endoplasmic reticulum of hippocampal neurons along with impaired spatial learning and memory abilities in mice (Chen et al. 2021). It has been previously reported that treatment of ovariectomized rats with a single dose of E2 increased the expression of the enzymes that comprise PDC in comparison to vehicle (Nilsen et al. 2007). In the present study, Pdha1 and Dlat levels were decreased in the Ovx + E2 group in relation to the Sham Group, reinforcing the participation of E2 in the regulation of energy substrate availability and mitochondrial structure and function in the long term.

Imbalances of the mitochondrial electron transport chain machinery were also observed in the present study. Cytochrome c oxidase (or complex IV), which is composed of 13 subunits, is the rate‐limiting enzyme of oxidative phosphorylation (OXPHOS). Administration of a single dose of E2 in ovariectomized rats increased both activity and expression of several subunits of cytochrome c oxidase (Irwin et al. 2008). The involvement of subunit Cox5a in age‐related memory impairment has been suggested in studies using a mice model displaying accelerated senescence, in which its overexpression improved memory and synaptic plasticity in the hippocampus (XiYang et al. 2016, 2020). In the present study, we found increased levels of Cox5a in the hippocampus of Ovx + E2 when compared to the Sham group. It is particularly noteworthy that decreased expression of stomatin‐like protein 2 (Stoml2) and sideroflexin‐3 (Sfxn3) was also observed in the Ovx + E2 group. Stoml2 binds to the mitochondrial inner membrane through the interaction with the glycerophospholipid cardiolipin forming mitochondrial membrane domains necessary for proper assembly and function of the respiratory chain, including Cox stabilization (Arnold and Kadenbach 1997). It has been shown that upregulation of Stoml2 increased mitochondrial biogenesis and function (Christie et al. 2011) and that its deficiency impaired mitochondrial translation (Mitsopoulos et al. 2017). Likewise, Sfxn3 is a protein also enriched in the inner mitochondrial membrane. Although it does not influence mitochondrial bioenergetics itself, it has been demonstrated that it influences synaptic plasticity (Amorim et al. 2017) and neurodegeneration (Ledahawsky et al. 2022). These changes further support the idea of the E2 influence on mitochondrial membrane organization and bioenergetics, which, in the long term, may influence menopause‐associated symptoms.

Acyl‐CoA thioesterases (Acots) play a key role in lipid metabolism and cellular signaling through the hydrolysis of fatty acyl‐CoAs into free fatty acids and coenzyme A. Acots' participation in the regulation of peroxisomal and mitochondrial fatty acyl‐CoA oxidation and in the subcellular trafficking of fatty acids has been suggested (Tillander et al. 2017). The cytosolic acyl coenzyme A thioester hydrolase, also called acyl‐CoA thioesterase 7 (Acot7), is the most abundant and active isoform found in the brain and it is highly expressed in the cytosol of neurons (Ellis et al. 2013). Knockout of Acot7 in mice brain induced defects in neuronal regulation of fatty acids metabolism under fasting conditions (such as increased free fatty acid, triacylglycerol, and phospholipid content), indicating that Acot7 might limit lipid accumulation in neurons in situations of free fatty acids oversupply (Ellis et al. 2013). The relationship between Acot7 and altered phospholipid profile has been shown in macrophages and in skeletal muscle of rats (Wall et al. 2017; Bakshi et al. 2018). The reduced levels of Acot7 in the hippocampus of Ovx + E2 group suggest derangements of lipid biosynthesis, which might interfere in both mitochondrial fatty acid oxidation and stability/dynamics of cellular membranes.

Besides its role in alcohol metabolism, aldehyde dehydrogenase 2 (Aldh2) also has a function in the oxidation of highly reactive aldehydes generated from endogenous metabolism of catecholamines and from lipid peroxidation induced by oxidative stress (Marchitti et al. 2007). Pieces of evidence suggest an association of obesity and metabolic syndrome with increased aldehyde products (Mattson 2009). Additionally, the involvement of Aldh2 and aldehydic load in neurodegeneration has been pointed out (Deza‐Ponzio et al. 2018; Joshi et al. 2019). A neuroprotective effect induced by the treatment with Alda‐1, an activator of Aldh2, of animal models of Alzheimer's disease and depression has been previously demonstrated (Stachowicz et al. 2016; Stachowicz et al. 2017; Mehder et al. 2020). Importantly, it has been observed that elderly diabetic patients presented ALDH2 mutations and had increased levels of both blood and urine levels of formaldehyde, which were strongly associated with cognitive deficits (Tan et al. 2018). Increased formaldehyde levels in the hippocampus of Aldh2 knockout mice were accompanied by cognitive impairment, and Aldh2 overexpression reduced formaldehyde levels and improved cognitive function (Tan et al. 2018; Ai et al. 2019). Since we found increased expression of Aldh2 in Ovx + E2 group in relation to the Sham group, it is reasonable to suggest that an antioxidant system involving the neutralization of aldehyde load is being recruited in ovariectomized rats after E2 replacement. Altered levels of glutathione S‐transferases (GSTs) and peroxiredoxins (Prdx), important players against oxidative stress, also strengthen the idea of activation of a protective mechanism against oxidative damage. In the present study, increased expression of Gstp1 in Ovx + E2 in relation to the Sham group, along with increased levels of Prdx2 in Ovx + E2, in relation to the Ovx group, were observed. It has been shown that aldehydes derived from lipid peroxidation enhance GST‐P expression in rat liver cells (Fukuda et al. 1997). Increased expression of Gstp1 has been observed in the hippocampus of aged rats (Li et al. 2020). Additionally, the involvement of Prdx2 against age‐related oxidative damage has been indicated (Kim et al. 2011).

The role of phosphatidylethanolamine‐binding protein 1 (Pebp1, also known as raf kinase inhibitory protein—RKIP—or precursor protein of hippocampal cholinergic neurostimulating peptide—HCNP‐pp), which induces acetylcholine synthesis, on both cognition and depression has been previously indicated. Stress exposure decreased the expression of Pebp1 in the hippocampus of male rats and impaired cognitive function (Kim and Kim 2007; Feldmann et al. 2008). Decreased levels of Pebp1 have also been observed in the hippocampus of T2D rats with mild cognitive impairment and pioglitazone treatment reversed these effects (Gao et al. 2017). Transgenic mice overexpressing Pebp1 in the hippocampus exhibited a depressive‐like phenotype in old age (Matsukawa et al. 2010). Conversely, antidepressant administration upregulated Pebp1 in the rat hippocampus (Khawaja et al. 2004). Interestingly, it has been suggested that Pebp1 participates in the regulation of vesicle trafficking and actin cytoskeleton organization (Schoentgen and Jonic 2020). The present dataset indicated that ovariectomy decreased the expression of Pebp1 and that E2 replacement exacerbated this response. The role of Pebp1 in the menopause associated symptoms deserves further investigation.

Taken together, the alterations on hippocampal proteins grouped in the metabolic processes cluster indicate the role of the estrogen milieu on mitochondrial function, oxidative stress and lipid metabolism, which may influence the structure and organization of membrane lipids, corroborating previous results related to menopause‐associated symptoms (Doshi and Agarwal 2013; Canerina‐Amaro et al. 2017; Sánchez‐Rodríguez et al. 2017; Zárate et al. 2017). These alterations have a close relationship with regulation of vesicle‐mediated transport and modulation of synaptic transmission, pathways that also showed statistical enrichment in the present study.

### 4.2 Significant Enrichment of Terms Related to Neuron Projection

The role of estrogen in cytoskeleton remodeling has been previously indicated (Unal et al. 2011; Sanchez et al. 2012). Among the altered proteins related to neuron projection, several take part in cytoskeleton organization. A role of microtubule‐associated protein 6 (Map6) in stabilization of actin filaments in dendritic spines and in regulating axonal elongation has been evidenced (Deloulme et al. 2015; Peris et al. 2018). Knockout of Map6 induces cognitive dysfunction such as memory impairment (Bégou et al. 2008). It has been indicated that alpha‐actinin‐1 (Actn1) participates in the regulation of Ca^2+^ influx into the cell through the control of surface localization and postsynaptic accumulation of the L‐type Ca^2+^ channel CaV1.2 which, in turn, mediates synaptic long‐term potentiation, controls neuronal excitability, and interacts with the β2 adrenergic receptor (Turner et al. 2020). Additionally, Actn1 also regulates the expression and function of the glutamate receptor type 5b on the cell surface, a mechanism dependent on the binding of Actn1 to the actin cytoskeleton (Cabello et al. 2007). Here, both Map6 and Actn1 presented enhanced expression in Ovx + E2 in relation to the Sham group. Additionally, the Ovx + E2 group showed an upregulation, in relation to the Sham group, of the neural cell adhesion molecule (Nrcam), a cell adhesion molecule found in the initial segments of axons which has a role in axon guidance and in the regulation of dendritic spine remodeling (Demyanenko et al. 2014). The role of these proteins in the hippocampus during menopause needs further investigations.

It has long been recognized that E2 modulates synaptic plasticity. In the present study, among the proteins participating in the modulation of chemical synaptic transmission, synaptophysin (Syp), vesicular glutamate transporter (Scl17a7) and glutamate receptor 1 (Gria1, also known as AMPA‐selective glutamate receptor 1or GluR1 or GluR‐A) deserve attention. Syp is an integral membrane protein component of synaptic vesicles and pieces of evidence suggest that it regulates synaptic vesicles trafficking and neurotransmitter release (Valtorta et al. 2004). Scl17a7 is responsible for vesicular storage and release of glutamate in synaptic vesicles, and it also activates Gria1 (Yu et al. 2018), which plays a crucial role in long term potentiation and learning (Mead and Stephens 2003). Activation of estrogen receptor beta increased synaptic proteins such as Syp and Gria1 and improved hippocampus‐dependent cognition (Liu et al. 2008). It has been shown that E2 administration to ovariectomized rats increased Syp expression along with increased dendritic spines only in the short term (McAsey et al. 2006; Velázquez‐Zamora et al. 2012). On the other hand, E2 replacement decreased Syp levels in the hippocampus of middle‐aged female rats (Williams et al. 2011). Accordingly, here we observed reduced levels of Syp in Ovx + E2 in relation to the Sham group. Otherwise, upregulation of Scl17a7 was detected in Ovx + E2 in relation to the Sham group while raised expression of Gria1 was observed in the Ovx group in comparison to the Sham group. These results point out to E2 influence on the glutamatergic synaptic transmission which may be associated with menopause‐associated symptoms such as memory impairments and mood disorders.

The results of the present study showed that E2 participates in the modulation of the expression of hippocampal proteins that take part in mitochondrial bioenergetics, lipid metabolism, vesicular trafficking and neuron projection. The present data point out the involvement of E2 in a cellular process where unbalances of energy metabolism and oxidative stress may affect the structure of vesicular membranes and, therefore, the cellular transport of molecules, hence, influencing synaptic transmission. The role of this system in the symptoms observed during menopause deserves deeper investigations. Additionally, the results indicated that E2 replacement at the dosage used in the present experiments was only able to partially reverse the ovariectomy‐induced alterations in the hippocampus. The proteins showing altered expression in the hippocampus, due to the absence and/or replacement of E2, as observed here, are possible novel therapeutic targets to prevent or ameliorate the postmenopause‐associated symptoms.

## Author Contributions

**Amanda Paula Pedroso:** conceptualization, methodology, writing – original draft, project administration, software, investigation. **Guilherme Araújo Câmara:** methodology, investigation. **Alexandre Keiji Tashima:** writing – review and editing, methodology, validation. **Lila Missae Oyama:** data curation, validation. **Eliane Beraldi Ribeiro:** conceptualization, supervision, writing – review and editing, resources, funding acquisition. **Valter Tadeu Boldarine:** methodology, software. **Adriana Pereira Souza:** writing – review and editing, data curation, visualization. **Meira Maria Forcellini Machado:** formal analysis, data curation. **Mônica Marques Telles:** writing – review and editing, validation.

## Funding

This work was supported by the Coordenação de Aperfeiçoamento de Pessoal de Nível Superior (CAPES–Finance Code 001 to A.P.P.); by the Conselho Nacional de Desenvolvimento Científico e Tecnológico (CNPq, grant 309505/2017‐8 to E.B.R.), and by Fundação de Amparo à Pesquisa do Estado de São Paulo (FAPESP, grant 2012/03172‐4 to E.B.R.).

## Ethics Statement

Animal protocols were approved by the Research Ethics Committee of the Universidade Federal de São Paulo—UNIFESP (#2172030315), according to the ethics guidelines settled by the Brazilian Conselho Nacional de Controle de Experimentação Animal (CONCEA).

## Conflicts of Interest

The authors declare no conflicts of interest.

## Supporting information


**Table S1:** Hippocampal proteome dataset (including proteins identified in at least four out of six biological replicates per group).
**Table S2:** Cellular location and biological processes of hippocampal proteins modulated by ovariectomy and estradiol replacement.
**Table S3:** Enrichment analysis of Gene Ontology (GO) terms, Kyoto Encyclopedia of Genes and Genomes (KEGG) pathways and Reactome pathways performed on STRING.

## Data Availability

The mass spectrometry proteomics data have been deposited to the ProteomeXchange Consortium (Deutsch et al. 2023) via the PRIDE partner repository (Perez‐Riverol et al. 2022) with the dataset identifier PXD040816.

## References

[jnc70536-bib-0001] Ai, L. , T. Tan , Y. Tang , et al. 2019. “Endogenous Formaldehyde Is a Memory‐Related Molecule in Mice and Humans.” Communications Biology 2: 1–12. 10.1038/s42003-019-0694-x. 31815201 PMC6884489

[jnc70536-bib-0002] Akhter, F. , D. Chen , A. Akhter , S. F. Yan , and S. D. Yan . 2021. “Age‐Dependent Accumulation of Dicarbonyls and Advanced Glycation Endproducts (AGEs) Associates With Mitochondrial Stress.” Free Radical Biology & Medicine 164: 429–438. 10.1016/j.freeradbiomed.2020.12.021. 33359687 PMC8552367

[jnc70536-bib-0003] AlAmmar, W. A. , F. H. Albeesh , and R. Y. Khattab . 2020. “Food and Mood: The Corresponsive Effect.” Current Nutrition Reports 9: 296–308. 10.1007/s13668-020-00331-3. 32623655

[jnc70536-bib-0004] Amin, Z. , T. Canli , and C. N. Epperson . 2005. “Effect of Estrogen‐Serotonin Interactions on Mood and Cognition.” Behavioral and Cognitive Neuroscience Reviews 4: 43–58. 10.1177/1534582305277152. 15886402

[jnc70536-bib-0005] Amorim, I. S. , L. C. Graham , R. N. Carter , et al. 2017. “Sideroflexin 3 Is an α‐Synuclein‐Dependent Mitochondrial Protein That Regulates Synaptic Morphology.” Journal of Cell Science 130: 325–331. 10.1242/jcs.194241. 28049716 PMC5278670

[jnc70536-bib-0006] Arnold, S. , and B. Kadenbach . 1997. “Cell Respiration Is Controlled by ATP, an Allosteric Inhibitor of Cytochrome‐c Oxidase.” European Journal of Biochemistry 249: 350–354. 10.1111/j.1432-1033.1997.t01-1-00350.x. 9363790

[jnc70536-bib-0007] Bakshi, I. , S. H. J. Brown , A. E. Brandon , et al. 2018. “Increasing Acyl CoA Thioesterase Activity Alters Phospholipid Profile Without Effect on Insulin Action in Skeletal Muscle of Rats.” Scientific Reports 8: 1–12. 10.1038/s41598-018-32354-w. 30228369 PMC6143561

[jnc70536-bib-0008] Bégou, M. , J. Volle , J.‐B. Bertrand , et al. 2008. “The Stop Null Mice Model for Schizophrenia Displays Cognitive and Social Deficits Partly Alleviated by Neuroleptics.” Neuroscience 157: 29–39. 10.1016/j.neuroscience.2008.07.080. 18804150

[jnc70536-bib-0009] Berent‐Spillson, A. , C. C. Persad , T. Love , et al. 2010. “Early Menopausal Hormone Use Influences Brain Regions Used for Visual Working Memory.” Menopause 17: 692–699. 10.1097/gme.0b013e3181cc49e9. 20300040 PMC2901395

[jnc70536-bib-0010] Boldarine, V. T. , E. Joyce , A. P. Pedroso , et al. 2021. “Oestrogen Replacement Fails to Fully Revert Ovariectomy‐Induced Changes in Adipose Tissue Monoglycerides, Diglycerides and Cholesteryl Esters of Rats Fed a Lard‐Enriched Diet.” Scientific Reports 11: 1–11. 10.1038/s41598-021-82837-6. 33589704 PMC7884784

[jnc70536-bib-0011] Boldarine, V. T. , A. P. Pedroso , C. Brandão‐Teles , et al. 2020. “Ovariectomy Modifies Lipid Metabolism of Retroperitoneal White Fat in Rats: A Proteomic Approach.” American Journal of Physiology. Endocrinology and Metabolism 319: E427–E437. 10.1152/ajpendo.00094.2020. 32663100

[jnc70536-bib-0012] Boldarine, V. T. , A. P. Pedroso , N. I. P. Neto , et al. 2019. “High‐Fat Diet Intake Induces Depressive‐Like Behavior in Ovariectomized Rats.” Scientific Reports 9: 1–9. 10.1038/s41598-019-47152-1. 31332243 PMC6646372

[jnc70536-bib-0013] Bredemann, T. M. , and L. L. McMahon . 2014. “17β Estradiol Increases Resilience and Improves Hippocampal Synaptic Function in Helpless Ovariectomized Rats.” Psychoneuroendocrinology 42: 77–88. 10.1016/j.psyneuen.2014.01.004. 24636504 PMC4065496

[jnc70536-bib-0014] Brinton, R. D. 2016. “Neuroendocrinology: Oestrogen Therapy Affects Brain Structure but Not Function.” Nature Reviews. Neurology 12: 561–562. 10.1038/nrneurol.2016.147. 27677967 PMC10128623

[jnc70536-bib-0015] Brownlee, M. 2005. “The Pathobiology of Diabetic Complications: A Unifying Mechanism.” Diabetes 54: 1615–1625. 10.2337/diabetes.54.6.1615. 15919781

[jnc70536-bib-0016] Cabello, N. , R. Remelli , L. Canela , et al. 2007. “Actin‐Binding Protein α‐Actinin‐1 Interacts With the Metabotropic Glutamate Receptor Type 5b and Modulates the Cell Surface Expression and Function of the Receptor.” Journal of Biological Chemistry 282: 12143–12153. 10.1074/jbc.M608880200. 17311919

[jnc70536-bib-0017] Canerina‐Amaro, A. , L. G. Hernandez‐Abad , I. Ferrer , et al. 2017. “Lipid Raft ER Signalosome Malfunctions in Menopause and Alzheimer's Disease.” Frontiers in Bioscience 9: 476. 10.2741/s476. 27814578

[jnc70536-bib-0018] Chen, W. , X. Sun , L. Zhan , W. Zhou , and T. Bi . 2021. “Conditional Knockout of Pdha1 in Mouse Hippocampus Impairs Cognitive Function: The Possible Involvement of Lactate.” Frontiers in Neuroscience 15: 1–11. 10.3389/fnins.2021.767560. PMC8552971 34720870

[jnc70536-bib-0019] Christie, D. A. , C. D. Lemke , I. M. Elias , et al. 2011. “Stomatin‐Like Protein 2 Binds Cardiolipin and Regulates Mitochondrial Biogenesis and Function.” Molecular and Cellular Biology 31: 3845–3856. 10.1128/mcb.05393-11. 21746876 PMC3165718

[jnc70536-bib-0020] Clegg, D. J. , L. M. Brown , S. C. Woods , and S. C. Benoit . 2006. “Gonadal Hormones Determine Sensitivity to Central Leptin and Insulin.” Diabetes 55: 978–987. 10.2337/diabetes.55.04.06.db05-1339. 16567519

[jnc70536-bib-0021] Davis, S. R. , I. Lambrinoudaki , M. Lumsden , et al. 2015. “Menopause.” Nature Reviews. Disease Primers 1: 1–19. 10.1038/nrdp.2015.4. 27188659

[jnc70536-bib-0022] Deloulme, J. C. , S. Gory‐Fauré , F. Mauconduit , et al. 2015. “Microtubule‐Associated Protein 6 Mediates Neuronal Connectivity Through Semaphorin 3E‐Dependent Signalling for Axonal Growth.” Nature Communications 6: 1–16. 10.1038/ncomms8246. PMC4468860 26037503

[jnc70536-bib-0023] Demyanenko, G. P. , V. Mohan , X. Zhang , et al. 2014. “Neural Cell Adhesion Molecule NrCAM Regulates Semaphorin 3F‐Induced Dendritic Spine Remodeling.” Journal of Neuroscience 34: 11274–11287. 10.1523/JNEUROSCI.1774-14.2014. 25143608 PMC4138338

[jnc70536-bib-0024] Deutsch, E. W. , N. Bandeira , Y. Perez‐Riverol , et al. 2023. “The ProteomeXchange Consortium at 10 Years: 2023 Update.” Nucleic Acids Research 51, no. D1: D1539–D1548. 36370099 10.1093/nar/gkac1040 PMC9825490

[jnc70536-bib-0025] Deza‐Ponzio, R. , M. L. Herrera , M. J. Bellini , M. B. Virgolini , and C. B. Hereñú . 2018. “Aldehyde Dehydrogenase 2 in the Spotlight: The Link Between Mitochondria and Neurodegeneration.” Neurotoxicology 68: 19–24. 10.1016/j.neuro.2018.06.005. 29936317

[jnc70536-bib-0026] Doncheva, N. T. , J. H. Morris , J. Gorodkin , and L. J. Jensen . 2019. “Cytoscape StringApp: Network Analysis and Visualization of Proteomics Data.” Journal of Proteome Research 18: 623–632. 10.1021/acs.jproteome.8b00702. 30450911 PMC6800166

[jnc70536-bib-0027] Doshi, S. B. , and A. Agarwal . 2013. “The Role of Oxidative Stress in Menopause.” Journal of Midlife Health 4: 140–146. 10.4103/0976-7800.118990. 24672185 PMC3952404

[jnc70536-bib-0028] Ellis, J. M. , G. W. Wong , and M. J. Wolfgang . 2013. “Acyl Coenzyme A Thioesterase 7 Regulates Neuronal Fatty Acid Metabolism to Prevent Neurotoxicity.” Molecular and Cellular Biology 33: 1869–1882. 10.1128/mcb.01548-12. 23459938 PMC3624175

[jnc70536-bib-0029] Fang, Y. Y. , P. Zeng , N. Qu , et al. 2018. “Evidence of Altered Depression and Dementia‐Related Proteins in the Brains of Young Rats After Ovariectomy.” Journal of Neurochemistry 146: 703–721. 10.1111/jnc.14537. 29939407

[jnc70536-bib-0030] Feldmann, R. E. , M. H. Maurer , C. Hunzinger , et al. 2008. “Reduction in Rat Phosphatidylethanolamine Binding Protein‐1 (PEBP1) After Chronic Corticosterone Treatment May Be Paralleled by Cognitive Impairment: A First Study.” Stress 11: 134–147. 10.1080/10253890701649904. 18311602

[jnc70536-bib-0031] Fritz, C. O. , P. E. Morris , and J. J. Richler . 2012. “Effect Size Estimates: Current Use, Calculations, and Interpretation.” Journal of Experimental Psychology. General 141: 2–18. 10.1037/a0024338. 21823805

[jnc70536-bib-0032] Fukuda, A. , Y. Nakamura , H. Ohigashi , T. Osawa , and K. Uchida . 1997. “Cellular Response to the Redox Active Lipid Peroxidation Products: Induction of Glutathione S‐Transferase P by 4‐Hydroxy‐2‐Nonenal.” Biochemical and Biophysical Research Communications 236: 505–509. 10.1006/bbrc.1997.6585. 9240470

[jnc70536-bib-0033] Gao, F. , L. Zang , D. Y. Wu , et al. 2017. “Pioglitazone Improves the Ability of Learning and Memory via Activating ERK1/2 Signaling Pathway in the Hippocampus of T2DM Rats.” Neuroscience Letters 651: 165–170. 10.1016/j.neulet.2017.04.052. 28458023

[jnc70536-bib-0034] Goto, M. , O. Abe , T. Miyati , et al. 2011. “3 Tesla MRI Detects Accelerated Hippocampal Volume Reduction in Postmenopausal Women.” Journal of Magnetic Resonance Imaging 33: 48–53. 10.1002/jmri.22328. 21182120

[jnc70536-bib-0035] Ha, D. M. , J. Xu , and J. S. Janowsky . 2007. “Preliminary Evidence That Long‐Term Estrogen Use Reduces White Matter Loss in Aging.” Neurobiology of Aging 28: 1936–1940. 10.1016/j.neurobiolaging.2006.08.007. 17030475 PMC2151930

[jnc70536-bib-0036] Hasegawa, Y. , Y. Hojo , H. Kojima , et al. 2015. “Estradiol Rapidly Modulates Synaptic Plasticity of Hippocampal Neurons: Involvement of Kinase Networks.” Brain Research 1621: 147–161. 10.1016/j.brainres.2014.12.056. 25595055

[jnc70536-bib-0037] Hill, D. A. , M. Crider , and S. R. Hill . 2016. “Hormone Therapy and Other Treatments for Symptoms of Menopause.” American Family Physician 94: 884–889. 27929271

[jnc70536-bib-0038] Hulsen, T. , J. de Vlieg , and W. Alkema . 2008. “BioVenn–A Web Application for the Comparison and Visualization of Biological Lists Using Area‐Proportional Venn Diagrams.” BMC Genomics 9: 1–6. 10.1186/1471-2164-9-488. 18925949 PMC2584113

[jnc70536-bib-0039] Huntley, R. P. , T. Sawford , P. Mutowo‐Meullenet , et al. 2015. “The GOA Database: Gene Ontology Annotation Updates for 2015.” Nucleic Acids Research 43: D1057–D1063. 10.1093/nar/gku1113. 25378336 PMC4383930

[jnc70536-bib-0040] Ialongo, C. 2016. “Understanding the Effect Size and Its Measures.” Biochemia Medica 26: 150–163. 10.11613/BM.2016.015. 27346958 PMC4910276

[jnc70536-bib-0041] Irwin, R. W. , J. Yao , R. T. Hamilton , E. Cadenas , R. D. Brinton , and J. Nilsen . 2008. “Progesterone and Estrogen Regulate Oxidative Metabolism in Brain Mitochondria.” Endocrinology 149: 3167–3175. 10.1210/en.2007-1227. 18292191 PMC2408802

[jnc70536-bib-0042] Jacobs, E. G. , B. K. Weiss , N. Makris , et al. 2016. “Impact of Sex and Menopausal Status on Episodic Memory Circuitry in Early Midlife.” Journal of Neuroscience 36: 10163–10173. 10.1523/JNEUROSCI.0951-16.2016. 27683911 PMC5039260

[jnc70536-bib-0043] Joshi, A. U. , L. D. van Wassenhove , K. R. Logas , et al. 2019. “Aldehyde Dehydrogenase 2 Activity and Aldehydic Load Contribute to Neuroinflammation and Alzheimer's Disease Related Pathology.” Acta Neuropathologica Communications 7: 1–18. 10.1186/s40478-019-0839-7. PMC6907112 31829281

[jnc70536-bib-0044] Kanoski, S. E. , and H. J. Grill . 2017. “Hippocampus Contributions to Food Intake Control: Mnemonic, Neuroanatomical, and Endocrine Mechanisms.” Biological Psychiatry 81: 748–756. 10.1016/j.biopsych.2015.09.011. 26555354 PMC4809793

[jnc70536-bib-0045] Kanoski, S. E. , M. R. Hayes , H. S. Greenwald , et al. 2011. “Hippocampal Leptin Signaling Reduces Food Intake and Modulates Food‐Related Memory Processing.” Neuropsychopharmacology 36: 1859–1870. 10.1038/npp.2011.70. 21544068 PMC3154104

[jnc70536-bib-0046] Khawaja, X. , J. Xu , J.‐J. Liang , and J. E. Barrett . 2004. “Proteomic Analysis of Protein Changes Developing in Rat Hippocampus After Chronic Antidepressant Treatment: Implications for Depressive Disorders and Future Therapies.” Journal of Neuroscience Research 75: 451–460. 10.1002/jnr.10869. 14743428

[jnc70536-bib-0047] Kim, H. G. , and K. L. Kim . 2007. “Decreased Hippocampal Cholinergic Neurostimulating Peptide Precursor Protein Associated With Stress Exposure in Rat Brain by Proteomic Analysis.” Journal of Neuroscience Research 85: 2898–2908. 10.1002/jnr.21407. 17628502

[jnc70536-bib-0048] Kim, S. U. , M. H. Jin , Y. S. Kim , et al. 2011. “Peroxiredoxin II Preserves Cognitive Function Against Age‐Linked Hippocampal Oxidative Damage.” Neurobiology of Aging 32: 1054–1068. 10.1016/j.neurobiolaging.2009.05.017. 19577336

[jnc70536-bib-0049] Kim, W. , H. J. Kwon , H. Y. Jung , D. Y. Yoo , D. W. Kim , and I. K. Hwang . 2020. “Phosphoglycerate Mutase 1 Reduces Neuronal Damage in the Hippocampus Following Ischemia/Reperfusion Through the Facilitation of Energy Utilization.” Neurochemistry International 133: 104631. 10.1016/j.neuint.2019.104631. 31836547

[jnc70536-bib-0050] Kucera, M. , R. Isserlin , A. Arkhangorodsky , and G. D. Bader . 2016. “AutoAnnotate: A Cytoscape App for Summarizing Networks With Semantic Annotations.” F1000Research 5: 1–12. 10.12688/f1000research.9090.1. PMC5082607 27830058

[jnc70536-bib-0051] Lam, T. K. T. 2007. “Brain Glucose Metabolism Controls Hepatic Glucose and Lipid Production.” Cell 3: 63–69. PMC2405765 18516245

[jnc70536-bib-0052] Ledahawsky, L. M. , M. E. Terzenidou , R. Edwards , et al. 2022. “The Mitochondrial Protein Sideroflexin 3 (SFXN3) Influences Neurodegeneration Pathways In Vivo.” FEBS Journal 289: 3894–3914. 10.1111/febs.16377. 35092170 PMC9542548

[jnc70536-bib-0053] Li, Y. , H. Yu , C. Chen , et al. 2020. “Proteomic Profile of Mouse Brain Aging Contributions to Mitochondrial Dysfunction, DNA Oxidative Damage, Loss of Neurotrophic Factor, and Synaptic and Ribosomal Proteins.” Oxidative Medicine and Cellular Longevity 2020: 1–21. 10.1155/2020/5408452. PMC7301248 32587661

[jnc70536-bib-0054] Lissner, L. J. , L. Rodrigues , K. M. Wartchow , et al. 2021. “Short‐Term Alterations in Behavior and Astroglial Function After Intracerebroventricular Infusion of Methylglyoxal in Rats.” Neurochemical Research 46: 183–196. 10.1007/s11064-020-03154-4. 33095439

[jnc70536-bib-0055] Liu, F. , M. Day , L. C. Muñiz , et al. 2008. “Activation of Estrogen Receptor‐β Regulates Hippocampal Synaptic Plasticity and Improves Memory.” Nature Neuroscience 11: 334–343. 10.1038/nn2057. 18297067

[jnc70536-bib-0056] Machado, M. M. F. , R. M. Banin , F. M. Thomaz , et al. 2021. “ *Ginkgo biloba* Extract (GbE) Restores Serotonin and Leptin Receptor Levels and Plays an Antioxidative Role in the Hippocampus of Ovariectomized Rats.” Molecular Neurobiology 58: 2692–2703. 10.1007/s12035-021-02281-5. 33492645

[jnc70536-bib-0057] Maki, P. M. , M. Wu , L. H. Rubin , et al. 2020. “Hot Flashes Are Associated With Altered Brain Function During a Memory Task.” Menopause 27: 269–277. 10.1097/GME.0000000000001467. 31913227

[jnc70536-bib-0058] Marchitti, S. A. , R. A. Deitrich , and V. Vasiliou . 2007. “Neurotoxicity and Metabolism of the Catecholamine‐Derived 3,4‐Dihydroxyphenylacetaldehyde and 3,4‐Dihydroxyphenylglycolaldehyde: The Role of Aldehyde Dehydrogenase.” Pharmacological Reviews 59: 125–150. 10.1124/pr.59.2.1. 17379813

[jnc70536-bib-0059] Margolis, K. L. , D. E. Bonds , R. J. Rodabough , et al. 2004. “Effect of Oestrogen Plus Progestin on the Incidence of Diabetes in Postmenopausal Women: Results From the Women's Health Initiative Hormone Trial.” Diabetologia 47: 1175–1187. 10.1007/s00125-004-1448-x. 15252707

[jnc70536-bib-0060] Matsukawa, N. , Y. Furuya , H. Ogura , and K. Ojika . 2010. “HCNP Precursor Protein Transgenic Mice Display a Depressive‐Like Phenotype in Old Age.” Brain Research 1349: 153–161. 10.1016/j.brainres.2010.06.041. 20599825

[jnc70536-bib-0061] Mattson, M. P. 2009. “Roles of the Lipid Peroxidation Product 4‐Hydroxynonenal in Obesity, the Metabolic Syndrome, and Associated Vascular and Neurodegenerative Disorders.” Experimental Gerontology 44: 625–633. 10.1016/j.exger.2009.07.003. 19622391 PMC2753676

[jnc70536-bib-0062] McAsey, M. E. , C. Cady , L. M. Jackson , et al. 2006. “Time Course of Response to Estradiol Replacement in Ovariectomized Mice: Brain Apolipoprotein E and Synaptophysin Transiently Increase and Glial Fibrillary Acidic Protein Is Suppressed.” Experimental Neurology 197: 197–205. 10.1016/j.expneurol.2005.09.008. 16226751

[jnc70536-bib-0063] McCarrey, A. C. , and S. M. Resnick . 2015. “Postmenopausal Hormone Therapy and Cognition.” Hormones and Behavior 74: 167–172. 10.1016/j.yhbeh.2015.04.018. 25935728 PMC4573348

[jnc70536-bib-0064] Mead, A. N. , and D. N. Stephens . 2003. “Selective Disruption of Stimulus‐Reward Learning in Glutamate Receptor gria1 Knock‐Out Mice.” Journal of Neuroscience 23: 1041–1048. 10.1523/jneurosci.23-03-01041.2003. 12574434 PMC6741941

[jnc70536-bib-0065] Mehder, R. H. , B. M. Bennett , R. D. Andrew , and S. Ferreira . 2020. “Morphometric Analysis of Hippocampal and Neocortical Pyramidal Neurons in a Mouse Model of Late Onset Alzheimer's Disease.” Journal of Alzheimer's Disease 74: 1069–1083. 10.3233/JAD-191067. PMC7242838 32144984

[jnc70536-bib-0066] Mitsopoulos, P. , O. Lapohos , W. Weraarpachai , H. Antonicka , Y. H. Chang , and J. Madrenas . 2017. “Stomatin‐Like Protein 2 Deficiency Results in Impaired Mitochondrial Translation.” PLoS One 12: 1–13. 10.1371/journal.pone.0179967. PMC5487072 28654702

[jnc70536-bib-0067] Monteleone, P. , G. Mascagni , A. Giannini , A. R. Genazzani , and T. Simoncini . 2018. “Symptoms of Menopause–Global Prevalence, Physiology and Implications.” Nature Reviews Endocrinology 14: 199–215. 10.1038/nrendo.2017.180. 29393299

[jnc70536-bib-0068] Nair, A. , and S. Jacob . 2016. “A Simple Practice Guide for Dose Conversion Between Animals and Human.” Journal of Basic and Clinical Pharmacy 7: 27–31. 10.4103/0976-0105.177703. 27057123 PMC4804402

[jnc70536-bib-0069] Naufel, M. F. , C. Frange , M. L. Andersen , et al. 2018. “Association Between Obesity and Sleep Disorders in Postmenopausal Women.” Menopause 25: 139–144. 10.1097/GME.0000000000000962. 28926516

[jnc70536-bib-0070] Naufel, M. F. , A. P. Pedroso , L. M. Oyama , M. M. Telles , H. Hachul , and E. B. Ribeiro . 2021. “Preliminary Evidence of Acylated Ghrelin Association With Depression Severity in Postmenopausal Women.” Scientific Reports 11: 1–9. 10.1038/s41598-021-84431-2. 33674672 PMC7935977

[jnc70536-bib-0071] Nilsen, J. , R. W. Irwin , T. K. Gallaher , and R. D. Brinton . 2007. “Estradiol In Vivo Regulation of Brain Mitochondrial Proteome.” Journal of Neuroscience 27: 14069–14077. 10.1523/JNEUROSCI.4391-07.2007. 18094246 PMC6673510

[jnc70536-bib-0072] Orosz, F. , J. Oláh , and J. Ovádi . 2006. “Triosephosphate Isomerase Deficiency: Facts and Doubts.” IUBMB Life 58: 703–715. 10.1080/15216540601115960. 17424909

[jnc70536-bib-0073] Pandey, R. , P. Shukla , B. Anjum , et al. 2020. “Estrogen Deficiency Induces Memory Loss via Altered Hippocampal HB‐EGF and Autophagy.” Journal of Endocrinology 244: 53–70. 10.1530/JOE-19-0197. 31648182

[jnc70536-bib-0074] Pascovici, D. , D. C. L. Handler , J. X. Wu , and P. A. Haynes . 2016. “Multiple Testing Corrections in Quantitative Proteomics: A Useful but Blunt Tool.” Proteomics 16: 2448–2453. 10.1002/pmic.201600044. 27461997

[jnc70536-bib-0075] Pedroso, A. P. , A. P. Souza , A. P. S. Dornellas , et al. 2017. “Intrauterine Growth Restriction Programs the Hypothalamus of Adult Male Rats: Integrated Analysis of Proteomic and Metabolomic Data.” Journal of Proteome Research 16: 1515–1525. 10.1021/acs.jproteome.6b00923. 28314371

[jnc70536-bib-0076] Pellerin, L. , and P. J. Magistretti . 2004. “Neuroscience. Let There be (NADH) Light.” Science 305: 50–52. 10.1126/science.1100428. 15232095

[jnc70536-bib-0077] Perez‐Riverol, Y. , J. Bai , C. Bandla , et al. 2022. “The PRIDE Database Resources in 2022: A Hub for Mass Spectrometry‐Based Proteomics Evidences.” Nucleic Acids Research 50, no. D1: D543–D552. 34723319 10.1093/nar/gkab1038 PMC8728295

[jnc70536-bib-0078] Peris, L. , M. Bisbal , J. Martinez‐Hernandez , et al. 2018. “A Key Function for Microtubule‐Associated‐Protein 6 in Activity‐Dependent Stabilisation of Actin Filaments in Dendritic Spines.” Nature Communications 9: 1–15. 10.1038/s41467-018-05869-z. PMC6141585 30224655

[jnc70536-bib-0079] Pratchayasakul, W. , N. Chattipakorn , and S. C. Chattipakorn . 2014. “Estrogen Restores Brain Insulin Sensitivity in Ovariectomized Non‐Obese Rats, but Not in Ovariectomized Obese Rats.” Metabolism 63: 851–859. 10.1016/j.metabol.2014.03.009. 24742706

[jnc70536-bib-0080] R Core Team . 2024. R: A Language and Environment for Statistical Computing. R Foundation for Statistical Computing. https://www.R‐project.org/.

[jnc70536-bib-0081] Sanchez, A. M. , M. I. Flamini , K. Polak , et al. 2012. “Actin Cytoskeleton Remodelling by Sex Steroids in Neurones.” Journal of Neuroendocrinology 24: 195–201. 10.1111/j.1365-2826.2011.02258.x. 22103470

[jnc70536-bib-0082] Sánchez‐Rodríguez, M. A. , L. Castrejón‐Delgado , M. Zacarías‐Flores , A. Arronte‐Rosales , and V. M. Mendoza‐Núñez . 2017. “Quality of Life Among Post‐Menopausal Women due to Oxidative Stress Boosted by Dysthymia and Anxiety.” BMC Women's Health 17: 1–9. 10.1186/s12905-016-0358-7. 28049464 PMC5209897

[jnc70536-bib-0083] Schoentgen, F. , and S. Jonic . 2020. “PEBP1/RKIP Behavior: A Mirror of Actin‐Membrane Organization.” Cellular and Molecular Life Sciences 77: 859–874. 10.1007/s00018-020-03455-5. 31960115 PMC11105014

[jnc70536-bib-0084] Shannon, P. , A. Markiel , O. Ozier , et al. 2003. “Cytoscape: A Software Environment for Integrated Models of Biomolecular Interaction Networks.” Genome Research 13: 2498–2504. 10.1101/gr.1239303. 14597658 PMC403769

[jnc70536-bib-0085] Silva, J. C. , R. Denny , C. A. Dorschel , et al. 2005. “Quantitative Proteomic Analysis by Accurate Mass Retention Time Pairs.” Analytical Chemistry 77: 2187–2200. 10.1021/ac048455k. 15801753

[jnc70536-bib-0086] Somogyi, V. , A. Gyorffy , T. J. Scalise , et al. 2011. “Endocrine Factors in the Hypothalamic Regulation of Food Intake in Females: A Review of the Physiological Roles and Interactions of Ghrelin, Leptin, Thyroid Hormones, Oestrogen and Insulin.” Nutrition Research Reviews 24: 132–154. 10.1017/S0954422411000035. 21418732

[jnc70536-bib-0087] Spencer, S. J. , A. Korosi , S. Layé , B. Shukitt‐Hale , and R. M. Barrientos . 2017. “Food for Thought: How Nutrition Impacts Cognition and Emotion.” NPJ Science of Food 1, no. 1: 7. 10.1038/s41538-017-0008-y. 31304249 PMC6550267

[jnc70536-bib-0088] Spencer‐Segal, J. L. , M. C. Tsuda , L. Mattei , et al. 2012. “Estradiol Acts via Estrogen Receptors Alpha and Beta on Pathways Important for Synaptic Plasticity in the Mouse Hippocampal Formation.” Neuroscience 202: 131–146. 10.1016/j.neuroscience.2011.11.035. 22133892 PMC3505616

[jnc70536-bib-0089] Stachowicz, A. , K. Głombik , R. Olszanecki , et al. 2016. “The Impact of Mitochondrial Aldehyde Dehydrogenase (ALDH2) Activation by Alda‐1 on the Behavioral and Biochemical Disturbances in Animal Model of Depression.” Brain, Behavior, and Immunity 51: 144–153. 10.1016/j.bbi.2015.08.004. 26254233

[jnc70536-bib-0090] Stachowicz, A. , R. Olszanecki , M. Suski , et al. 2017. “Proteomic Analysis of Mitochondria‐Enriched Fraction Isolated From the Frontal Cortex and Hippocampus of Apolipoprotein e Knockout Mice Treated With Alda‐1, an Activator of Mitochondrial Aldehyde Dehydrogenase (ALDH2).” International Journal of Molecular Sciences 18: 1–16. 10.3390/ijms18020435. PMC5343969 28218653

[jnc70536-bib-0091] Su, J. , K. Sripanidkulchai , Y. Hu , J. M. Wyss , and B. Sripanidkulchai . 2012. “The Effect of Ovariectomy on Learning and Memory and Relationship to Changes in Brain Volume and Neuronal Density.” International Journal of Neuroscience 122: 549–559. 10.3109/00207454.2012.690795. 22578066

[jnc70536-bib-0092] Suarez, A. N. , E. E. Noble , and S. E. Kanoski . 2019. “Regulation of Memory Function by Feeding‐Relevant Biological Systems: Following the Breadcrumbs to the Hippocampus.” Frontiers in Molecular Neuroscience 12: 1–21. 10.3389/fnmol.2019.00101. 31057368 PMC6482164

[jnc70536-bib-0093] Suzuki, A. , S. A. Stern , O. Bozdagi , et al. 2011. “Astrocyte‐Neuron Lactate Transport Is Required for Long‐Term Memory Formation.” Cell 144: 810–823. 10.1016/j.cell.2011.02.018. 21376239 PMC3073831

[jnc70536-bib-0094] Tajes, M. , B. Guivernau , E. Ramos‐Fernández , et al. 2013. “The Pathophysiology of Triose Phosphate Isomerase Dysfunction in Alzheimer's Disease.” Histology and Histopathology 28: 43–51. 10.14670/HH-28.43. 23233058

[jnc70536-bib-0095] Tan, T. , Y. Zhang , W. Luo , et al. 2018. “Formaldehyde Induces Diabetes‐Associated Cognitive Impairments.” FASEB Journal 32: 3669–3679. 10.1096/fj.201701239R. 29401634

[jnc70536-bib-0096] Thaung Zaw, J. J. , P. R. C. Howe , and R. H. X. Wong . 2018. “Postmenopausal Health Interventions: Time to Move on From the Women's Health Initiative?” Ageing Research Reviews 48: 79–86. 10.1016/j.arr.2018.10.005. 30355506

[jnc70536-bib-0097] Tillander, V. , S. E. H. Alexson , and D. E. Cohen . 2017. “Deactivating Fatty Acids: Acyl‐CoA Thioesterase‐Mediated Control of Lipid Metabolism.” Trends in Endocrinology and Metabolism 28: 473–484. 10.1016/j.tem.2017.03.001. 28385385 PMC5474144

[jnc70536-bib-0098] Turner, M. , D. E. Anderson , P. Bartels , et al. 2020. “α‐Actinin‐1 Promotes Activity of the L‐Type Ca^2+^ Channel ca v 1.2.” EMBO Journal 39: 1–21. 10.15252/embj.2019102622. PMC7507692 33433005

[jnc70536-bib-0099] Unal, D. , Z. Halici , Z. Altunkayna , O. N. Keles , E. Oral , and B. Unal . 2011. “A New Hypothesis About Neuronal Degeneration Appeared After a Rat Model of Menopause.” Neurodegenerative Diseases 9: 25–30. 10.1159/000329721. 21876334

[jnc70536-bib-0100] Valtorta, F. , M. Pennuto , D. Bonanomi , and F. Benfenati . 2004. “Synaptophysin: Leading Actor or Walk‐On Role in Synaptic Vesicle Exocytosis?” BioEssays 26: 445–453. 10.1002/bies.20012. 15057942

[jnc70536-bib-0101] Velázquez‐Zamora, D. A. , D. González‐Tapia , M. M. González‐Ramírez , et al. 2012. “Plastic Changes in Dendritic Spines of Hippocampal CA1 Pyramidal Neurons From Ovariectomized Rats After Estradiol Treatment.” Brain Research 1470: 1–10. 10.1016/j.brainres.2012.06.012. 22750586

[jnc70536-bib-0102] Wall, V. Z. , S. Barnhart , F. Kramer , et al. 2017. “Inflammatory Stimuli Induce Acyl‐CoA Thioesterase 7 and Remodeling of Phospholipids Containing Unsaturated Long (≥ C20)‐Acyl Chains in Macrophages.” Journal of Lipid Research 58: 1174–1185. 10.1194/jlr.M076489. 28416579 PMC5454516

[jnc70536-bib-0103] Weber, M. T. , M. Mapstone , J. Staskiewicz , and P. M. Maki . 2012. “Reconciling Subjective Memory Complaints With Objective Memory Performance in the Menopausal Transition.” Menopause 19: 735–741. 10.1097/gme.0b013e318241fd22. 22415562 PMC3773730

[jnc70536-bib-0104] Williams, T. J. , K. L. Mitterling , L. I. Thompson , et al. 2011. “Age‐ and Hormone‐Regulation of Opioid Peptides and Synaptic Proteins in the Rat Dorsal Hippocampal Formation.” Brain Research 1379: 71–85. 10.1016/j.brainres.2010.08.103. 20828542 PMC3020269

[jnc70536-bib-0105] Xiyang, Y. B. , R. Liu , X. Y. Wang , et al. 2020. “COX5A Plays a Vital Role in Memory Impairment Associated With Brain Aging via the BDNF/ERK1/2 Signaling Pathway.” Frontiers in Aging Neuroscience 12: 1–13. 10.3389/fnagi.2020.00215. 32754029 PMC7365906

[jnc70536-bib-0106] XiYang, Y. B. , Y. C. Wang , Y. Zhao , et al. 2016. “Sodium Channel Voltage‐Gated Beta 2 Plays a Vital Role in Brain Aging Associated With Synaptic Plasticity and Expression of COX5A and FGF‐2.” Molecular Neurobiology 53: 955–967. 10.1007/s12035-014-9048-3. 25575679

[jnc70536-bib-0107] Yu, H. , M. Li , D. Zhou , et al. 2018. “Vesicular Glutamate Transporter 1 (VGLUT1)‐Mediated Glutamate Release and Membrane GluA1 Activation Is Involved in the Rapid Antidepressant‐Like Effects of Scopolamine in Mice.” Neuropharmacology 131: 209–222. 10.1016/j.neuropharm.2017.12.028. 29274366

[jnc70536-bib-0108] Zárate, S. , M. Astiz , N. Magnani , et al. 2017. “Hormone Deprivation Alters Mitochondrial Function and Lipid Profile in the Hippocampus.” Journal of Endocrinology 233: 1–14. 10.1530/JOE-16-0451. 28130408

[jnc70536-bib-0109] Zhao, L. , M. Dong , M. Ren , C. Li , H. Zheng , and H. Gao . 2018. “Metabolomic Analysis Identifies Lactate as an Important Pathogenic Factor in Diabetes‐Associated Cognitive Decline Rats.” Molecular & Cellular Proteomics 17: 2335–2346. 10.1074/mcp.RA118.000690. 30171160 PMC6283288

[jnc70536-bib-0110] Zhao, L. , Y. Jia , D. Yan , C. Zhou , J. Han , and J. Yu . 2013. “Aging‐Related Changes of Triose Phosphate Isomerase in Hippocampus of Senescence Accelerated Mouse and the Intervention of Acupuncture.” Neuroscience Letters 542: 59–64. 10.1016/j.neulet.2013.03.002. 23499955

